# Wearable and Nearable Biosensors and Systems for Healthcare

**DOI:** 10.3390/s21041291

**Published:** 2021-02-11

**Authors:** Marco Di Rienzo, Ramakrishna Mukkamala

**Affiliations:** 1 Polo Tecnologico, IRCCS Fondazione Don Carlo Gnocchi ONLUS, 20148 Milano, Italy; 2 Department of Bioengineering and Department of Anesthesiology and Perioperative Medicine, University of Pittsburgh, Pittsburgh, PA 15261, USA; rmukkamala@pitt.edu

Biosensors and systems in the form of wearables and “nearables” (i.e., everyday sensorized objects with transmitting capabilities such as smartphones) are rapidly evolving for use in healthcare. Unlike conventional approaches, these technologies can enable seamless or on-demand physiological monitoring anytime and anywhere. Such monitoring can be beneficial in various ways. Most notably, it can help transform healthcare from the current reactive, one-size-fits-all, hospital-centered, and volume-based system into a future proactive, personalized, decentralized, and valued-based system. This new system and other benefits of the technology hold great promise for longer and healthier living.

Wearable and nearable biosensors and systems have been made possible through integrated innovations in sensor design, electronics, data transmission, power management, and signal processing. Examples of measurements offered by these technologies include biopotentials, body motion, pressure, blood volume, temperature, and biochemical markers. Although much progress has been made in this field, many open challenges for the scientific community remain, especially for those applications requiring high accuracy.

The aim of this Special Issue of *Sensors* is to provide an open collection of state-of-the-art investigations on wearable and nearable biosensors and systems in order to foster further technological advances and the use of the technology to benefit healthcare. The 12 papers that constitute this Special Issue offer both depth and breadth pertaining to wearable and nearable technology [1,2,3,4,5,6,7,8,9,10,11,12]. Depth is afforded through a critical mass of studies on accelerometers [3,4,5,7,9,10], signal processing [1,2,4,5,7,10,11], and cardiovascular monitoring applications [2,5,6,7,9,10,12], whereas breadth is given through new biosensors [12] and data transmission [9], other clinical applications including surgical training [8] and brain-computer interfaces [1], and validation of commercial devices [6], which is crucial for adoption. We provide a flavor of each contribution below in order of appearance in this issue.

Majidov et al. [1] developed a machine learning technique to analyze EEG signals from a wearable electrode cap for brain–computer interface (BCI) applications. The technique was developed using a formal BCI competition dataset in which 18 subjects performed an imaginary movement of hands and feet, and comprised a number of analytical tools including online deep learning with data augmentation. They showed that the technique was able to increase the classification accuracy compared to earlier techniques.

Huysmans et al. [2] developed a machine learning technique to analyze a bed-based ballistocardiography (BCG) signal (i.e., a measure of the whole-body movement induced by the heartbeat) for sleep apnea screening and sleep monitoring. The fully automatic technique employed unsupervised, k-means clustering to reveal artifact (apnea) versus clean signals. They showed that the technique can detect subjects with significant apnea while also revealing how the bed pressure sensor should be used to enable future supervised learning.

Bolus et al. [3] developed a glove with a fingertip mounted accelerometer for monitoring the health of joints via acoustic emissions. The new form factor for measuring joint sounds eliminates the need for consumables like tape and associated interface noise. They showed that the device can yield reliable measurements under constant fingertip contact force in subjects during an intervention to alter the knee joint sound.

Del Rosario et al. [4] developed a machine learning technique to determine body position from a smartphone inertial measurement unit (IMU) placed at an arbitrary orientation for various applications including fall detection. The technique uses hand-crafted instead of deep-learning-based IMU features learned during walking periods as a reference for upright body posture. They showed that this technique can separate standing versus sedentary periods using only one smartphone IMU in the pocket of younger and older subjects. 

Yao et al. [5] developed a wearable system for cuff-less tracking of blood pressure changes. The system measures a BCG signal via an armband accelerometer and a photo-plethysmography (PPG) signal via a finger clip, extracts data-driven features including the time delay between the signals (pulse transit time), and performs cuff calibration to map the features to blood pressure. They showed that this system as well as a BCG–PPG weighing scale system could track blood pressure changes during interventions in healthy subjects. 

Passier et al. [6] performed a validation study to compare two commercial in-ear PPG sensor devices for detecting heart rate during intense physical activity. The study is unique in terms of assessing external auditory canal PPG sensor devices and included 20 subjects during graded cycling. Both devices attained acceptable mean absolute heart rate errors compared to the reference ECG over a wide heart rate range but were not particularly precise.

Landreani et al. [7] developed a signal processing technique to quantify ultra-short-term heart rate variability via a smartphone accelerometer for stress detection. The technique involves placing the smartphone on the abdomen and detecting each heartbeat from the resulting BCG signal via cross correlation with a template. They showed the efficacy of the technique in detecting vagal withdrawal during mental arithmetic in healthy subjects.

de Mathelin et al. [8] developed a glove for establishing objective criteria of the expertise needed for surgeons to operate a transluminal robotic assistance system. The glove includes 12 wireless force sensitive resistors for measuring hand grip forces under visual feedback from the system. They revealed important differences in the handgrip forces of an expert versus a novice in performing an exemplary pick and drop task. 

Di Rienzo et al. [9] developed a wearable acquisition platform for the monitoring of various cardiovascular features including pulse transit time and cardiac contractility. The platform is capable of measuring 36 signals from 12 wireless nodes, including ECG, seismocardiography (SCG, i.e., accelerometer-based measure of chest vibrations caused by the heartbeat), and PPG sensors. Field tests showed that the system can acquire good quality data in real life with a synchronization error between nodes lower than 1ms. 

Yu et al. [10] developed a signal processing technique to remove the common motion artifact in the SCG signal. Since the artifact is typically mixed with the heartbeat in time and frequency, the technique is based on adaptive recursive least squares. They showed that the technique could extract a clear signal without further processing from only one accelerometer and detect the heart rate with high accuracy in healthy subjects. 

Asci et al. [11] developed a machine learning technique to analyze smartphone voice signals for assessing physiological aging. The technique extracts thousands of signal features, performs feature reduction, and then applies a support vector machine to classify age and gender. They notably showed the efficacy of the technique in subjects in a free-living scenario to eliminate potential voice changes in supervised conditions. 

Farooq et al. [12] developed a thin-filmed flexible wireless pressure sensor for interface pressure monitoring during leg compression treatment of venous insufficiency. The sensor is based on a pressure-dependent capacitance and inductive coil to allow passive and wireless measurement. Through analytical and experimental testing, they showed that the sensor offers sensitivity that is competitive with existing technology but with lower-cost fabrication.

We hope that this editorial serves as a useful guide to this collection of papers and that the Special Issue does end up inspiring future efforts to bring an array of wearable and nearable biosensors and systems to healthcare practices.

## Data Availability

Not applicable.
